# The Chain Distribution Tensor: Linking Nonlinear Rheology and Chain Anisotropy in Transient Polymers

**DOI:** 10.3390/polym10080848

**Published:** 2018-08-01

**Authors:** Shankar Lalitha Sridhar, Franck J. Vernerey

**Affiliations:** 1 Department of Mechanical Engineering, University of Colorado Boulder, Boulder, CO 80309, USA; shla6400@colorado.edu; 2 Materials Science and Engineering Program, University of Colorado Boulder, Boulder, CO 80309, USA

**Keywords:** shear thickening, nonlinear rheology, polymer mechanics, supramolecular polymers, associative polymers, transient network theory, dynamic bond

## Abstract

Transient polymer networks are ubiquitous in natural and engineered materials and contain cross-links that can reversibly break and re-form. The dynamic nature of these bonds allows for interesting mechanical behavior, some of which include nonlinear rheological phenomena such as shear thickening and shear thinning. Specifically, physically cross-linked networks with reversible bonds are typically observed to have viscosities that depend nonlinearly on shear rate and can be characterized by three flow regimes. In slow shear, they behave like Newtonian fluids with a constant viscosity. With further increase in shear rate, the viscosity increases nonlinearly to subsequently reach a maximum value at the critical shear rate. At this point, network fracture occurs followed by a reduction in viscosity (shear-thinning) with a further increase in shear rate. The underlying mechanism of shear thickening in this process is still unclear with debates between a conversion of intra-chain to inter-chain cross-linking and nonlinear chain stretch under high tension. In this paper, we provide a new framework to describe the nonlinear rheology of transient polymer networks with the so-called chain distribution tensor using recent advances from the transient network theory. This tensor contains quantitatively and statistical information of the chain alignment and possible anisotropy that affect network behavior and mechanics. We investigate shear thickening as a primary result of non-Gaussian chain behavior and derive a relationship for the nonlinear viscosity in terms of the non-dimensional Weissenberg number. We further address the criterion for network fracture at the critical shear rate by introducing a critical chain force when bond dissociation is suddenly accelerated. Finally, we discuss the role of cross-linker density on viscosity using a “sticky” reptation mechanism in the context of previous studies on metallo-supramolecular networks with reversible cross-linkers.

## 1. Introduction

Transient polymer networks are characterized by dynamic bonding or cross-linking between polymer chains that reversibly break and reform [1,2,3]. Most natural materials that aid in performing some of the key functions of life such as growth and self-repair are indeed dynamic and are often composed of transient biopolymer networks. As a result of their dynamic bonds, these networks often exhibit complex mechanical behaviors wherein they can transition between a solid state (that provides mechanical strength) and a fluid state (that flows by structural reorganization) [4]. Plant and fungal cell walls are prime examples of such behavior as they are composed of a transient polymer network of polysaccharides and proteins [5]. This enables an otherwise sturdy wall structure to morph in response to stimuli such as light or gravity and more importantly during growth and self-repair. Transient polymer networks also form the chemical engine of life to generate motion such as muscle cells made up actin–myosin assemblies that walk powered by ATP to cause muscle contraction [6]. Mechanosensitivity is prevalent in many types of cells that rely on adaptable active bio-polymer networks [7]. Today, synthetic polymers are being widely studied to engineer reversible intermolecular interactions that lead to multi-functional abilities such as self-healing, stimuli-responsiveness and shape memory [8,9,10] that have found many engineering applications including in biomedicine through tissue engineering [11,12,13]. These polymers are typically synthesized by using non-covalent bonds such as hydrogen bonds, ionic or dipolar bonds, or metal-ligand bonds, between polymer chains and have been categorized as supramolecular polymers [14,15,16]. They are found to have rich viscoelastic properties that are arguably enhanced (longer relaxation time, higher modulus plateau, etc.) compared to their covalent counterparts that achieve flow by polymer chain diffusion (unentangled) or reptation (entangled) [17,18,19,20,21]. Furthermore, supramolecular polymers exhibit interesting nonlinear rheological behaviors such as shear thickening and strain hardening whose key mechanisms are not that well understood. These interesting nonlinear properties can potentially be exploited for energy dissipation in military applications such as body armors where the shear thickening property may prove highly useful [22].

Newtonian fluids are characterized by a viscosity that remains constant for any applied shear rate. In contrast, shear thickening fluids are characterized by a non-Newtonian behavior where the viscosity increases nonlinearly with shear rate. In other words, the tendency of the fluid to flow degenerates under stress, which is quite contrary to commonly seen non-Newtonian behavior where the tendency to flow improves under stress (shear-thinning) [23]. Interestingly transient polymer networks such as metallo-supramolecular and telechelic associative polymers [24,25,26,27,28] are commonly observed to have three different flow regimes. At low shear rates, the behavior is similar to that of a Newtonian fluid. At a critical rate, shear thickening is initiated which continues until reaching a maximum viscosity at which point the final regime of shear thinning is initiated. The molecular origin of the shear thickening response in transient polymer networks has been the topic of much debate among both experimental and theoretical researchers. The governing mechanism can be categorized broadly by two schools of thought namely, (a) an increased number of stretched polymer chains with applied shear [29,30,31,32,33], and (b) a nonlinear high tension in chains beyond the Gaussian range at large stretch [34,35,36]. The first mechanism (a) attributes shear thickening to the structural re-organization of the polymer network that increases the number of chains that are under tension. This is argued to be a result of shear induced conversion of free chains and linking within the same chain to cross-linking between different chains [29,33,37]. This conversion is caused by extended chain configurations at large flow rates that make intra-chain connections less favorable than inter-chain cross-linking. However, Wang et al [32] argued against this mechanism claiming that intra-chain connections cannot be broken before inter-chain cross-links, when bond lifetime is longer than the relaxation time of free chains with intra-chain connections. Subsequently, a model was proposed where shear-thickening was predicted as a result of coagulation of free chains in an existing polymer network. The second mechanism (b) is based on the idea that shear thickening occurs because of polymer chains that harden from overstretching, also called non-Gaussian chain stretching. Marucci [34] showed that shear thickening can be predicted from just nonlinear chain stretching by keeping the number of stretched chains constant during shear by proposing the so-called free path model. While this model has received some support from experimental measurements of critical shear rate and stress (the point of onset shear thickening), it is argued that it is not a direct proof of cause as the first mechanism also has similar dynamic signatures (chain orientation under shear) [29]. A general quantitative treatment of nonlinear rheology, including shear thickening and thinning, has been given by previous researchers using the transient network theory with a statistical chain description [34,38,39] albeit mathematically involved. An elegant approach to describing these nonlinear rheological phenomena with information on the statistical properties and resulting anisotropy of the chain orientations during flow is still lacking.

The objective of this paper is to contribute towards the understanding of nonlinear rheology, specifically shear thickening, using the transient network theory [2,40,41] to provide statistical and quantitative information on the chain length, orientation and stretch during shear. For this purpose, we define the chain distribution tensor, interpreted as the covariance matrix of chain end-to-end vectors, from our recent work in transient network theory, that will be a key element to a better understanding and visualization of the chain population behavior [41,42]. The novelty of this work is in the simple relationships for nonlinear rheology based on chain properties and bond lifetimes of cross-linkers that have remained difficult so far. While approaches such as coarse-grained molecular dynamics provide a methodolgy to investigate molecular mechanisms in these networks, it is often computationally expensive and difficult to obtain clear theoertical relationships to macroscopic properties. The article is organized as follows. We first provide the governing stochastic equations from the transient network theory (TNT) with its most recent advances that correspond to the chain distribution tensor. This is followed by the application of TNT to address three main phenomena, (a) nonlinear viscosity at different types of steady flows, (b) network fracture at a critical shear rate, and (c) the role of cross-link concentration in the viscosity of the polymer network in the context of studies on metallo-supramolecular networks with reversible cross-linking from the literature.

## 2. Transient Network Theory for Polymer Networks with Reversible Bonds

We first review some of the key aspects and equations of the Transient Network Theory (TNT) for dynamic polymers by introducing concepts from recent advances in this field of study [41,42].

### 2.1. Statistical Description of Polymer Chains.

**Figure 1:**
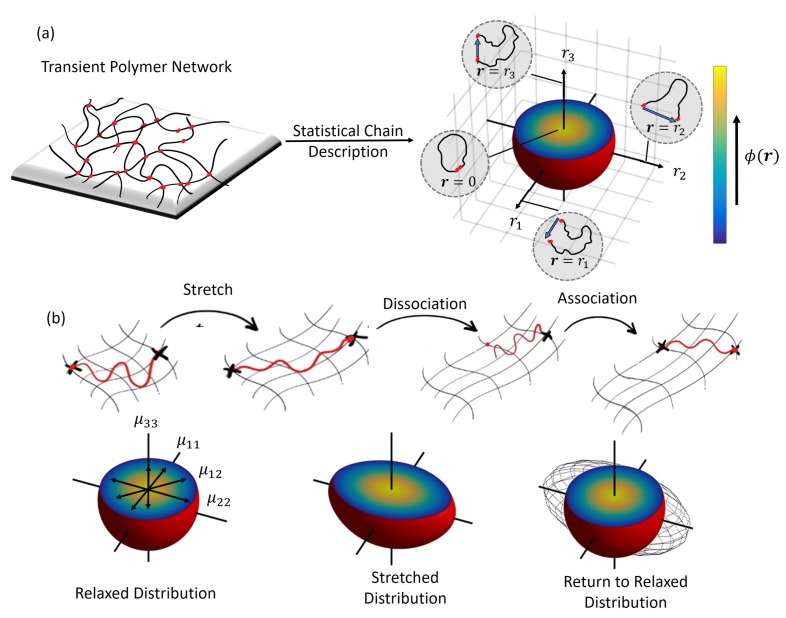
Statistical description of a transient polymer network with (**a**) the end-to-end vector, r and the chain distribution function, ϕ(r) illustrated with 3D mapping, and (**b**) evolution of the chain distribution function, ϕ(r) and tensor μ shown w.r.t the chain stretch λ.

For simplicity, the chains of the polymer network are assumed to be long enough such that they are never fully straightened. This allows us to follow Gaussian statistics for chain end-to-end distances and force-independent bond kinetics for the reversible cross-links. The states of the polymer chains and their contribution in the network describe the overall mechanical response of the material. For instance, in an isotropic network, the chain population spans all the directions and can be suitably described by their end-to-end vector, r that contains information of both length, r=|r|, and direction (polar angle θ and azimuthal angle ω in spherical coordinates). The statistical description of the chain population is then given by the chain distribution function, ϕ(r)=ϕ(r,θ,ω), that can be used to describe the mechanics of the network of chains within a continuum point (Figure 1a). Let the chain stretch ratio be defined as $\lambda =r/r_{0}$, where $r_{0}=\sqrt{Nb^{2}/3}$ is the average length of a polymer chain with *N* Kuhn segments of length *b* at stress-free state. The distribution function provides two important quantities: the density of chains attached to the network, *c*, and the so-called chain distribution tensor (μ) given by

(1a) $$\begin{array}{ccc} c & = & \int_{\Omega }\phi \left(r\right)d\Omega , \end{array}$$

(1b) $$\begin{array}{ccc} \mu & = & \frac{1}{c}\int_{\Omega }\phi \left(r\right)\;3\;\left(\lambda ⊗\lambda \right)\;d\Omega , \end{array}$$

where ⊗ denotes tensor product and Ω represents the space of all possible chain configurations. The tensor μ is symmetric and represents, in an average sense, the stretch (direction and magnitude) experienced by chains in the polymer network [41]. In fact, due to its definition, it may also be interpreted as the covariance matrix of the directional components of the chain end-to-end stretch ratio, λ. The principal values of this tensor, $\mu_{1}$, $\mu_{2}$ and $\mu_{3}$ can then be physically interpreted as the standard deviations of the end-to-end stretch ratio, λ of chains in the corresponding principal directions. Therefore, relevant measures of the physical state of the network such as the root mean square of chain stretch, $\lambda_{rms}$, can be obtained from the distribution tensor as follows:

(2) $$\lambda_{rms}=\sqrt{\frac{tr(\mu )}{3}}=\sqrt{\frac{\mu_{1}+\mu_{2}+\mu_{3}}{3}}.$$

It is easy to show from Equations (1b) and (2) that when the polymer network is stress-free, the chain stretch is given by $\lambda =1/\sqrt{3}\;\left(1,1,1\right),$ which results in μ=I and $\lambda_{rms}=1$. Furthermore, μ is also directly related to the stored elastic energy in the polymer network as we will show below.

### 2.2. Evolution equations.

The change in the state of the chain population in time can be characterized by determining the evolution of the chain distribution function in time. For this purpose, consider a small material volume of the polymer network undergoing deformation over time. The distribution of attached chains is affected by three physical processes (Figure 1b):

(a) The rate of change of chain stretch, $\dot{\lambda }$, due to network deformation that is governed by the macroscopic velocity gradient L=∇v, where: v is the velocity field in the material and ∇ is the differential operator. If we assume that the chains undergo affine deformation (i.e., they follow the macroscopic deformation L), the stretch rate is given by $\dot{\lambda }=L\lambda$.
(b) The association of new chains into the network with rate, $k_{a}$, at a near stretch-free configuration that follows the well-known Gaussian probability density function for polymer chains, $p_{0}\left(r\right)$.
(c) The dissociation of attached chains that may be in a stretched state at the rate $k_{d}$.

Therefore, the evolution of the chain population in terms of the distribution function, ϕ(r,t) and the distribution tensor, μ(t) are given by [42]:

(3a) $$\begin{array}{c} \dot{\phi }=-L:\left(\nabla \phi ⊗\lambda \right)+k_{a}\left(C-c\right)p_{0}-k_{d}\phi , \end{array}$$

(3b) $$\begin{array}{c} \overset{\circ }{\mu }=k_{a}\left(\frac{C-c}{c}\right)I-k_{d}\mu -\frac{\dot{c}}{c}\mu , \end{array}$$

where ∇ϕ=∂ϕ/∂λ, and $\overset{\circ }{\mu }=\dot{\mu }-\left(L\mu +\mu L^{T}\right)$ is the Truesdell rate, an objective tensor rate defined typically for the Kirchoff stress tensor. *C* is the total number of linear polymer chains which is unchanging in time due to incompressibility of the polymer network. We note here that the general evolution equation of the tensor μ is simplified to Equation (3b) under the assumption that the dissociation rate $k_{d}$ is independent of chain tension [41]. We further note that mechanisms of chain conformation dynamics in non-entangled polymer solutions such as that described by the elastic dumbbell and Rouse models, which involve the elastic energy of chains, can be used to augment the evolution of μ [23]. This is out of the scope of the current paper and is left as the object of future studies. The density of attached chains, *c*, is unaffected by the deformation history and depends only the association and dissociation terms of Equation (3b) as

(4) $$\dot{c}=k_{a}\left(C-c\right)-k_{d}c.$$

When the rate of chain association and dissociation are constants, the concentration of attached chains is decoupled from deformation and remains in the steady state (i.e., $\dot{c}=$ 0). This means that the density of attached chains becomes, $c=Ck_{a}/\left(k_{a}+k_{d}\right)$. In this work, for simplicity, we neglect entropic chain relaxations described by the Zimmm and Rouse models. The framework may still be used to include these effects by defining another term in the evolution equation of the chain population in Equation (3a) that affects the end-to-end distances of the chain due to such relaxation mechanisms.

### 2.3. Macroscopic energy and stress

The statistical description of chains can be used to derive macroscopic mechanical entities of the network such as stored elastic energy, dissipation energy and the stress tensor. The stored elastic energy can be obtained from the difference between the deformed state and the stress-free state in terms of the distribution tensor as

(5) $$\Delta \Psi_{e}=ck_{B}T\left[\frac{1}{2}\left(tr\left(\mu \right)-3\right)+\frac{1}{20N}\left(tr{\left(\mu \right)}^{2}-9\right)+\frac{11}{1150N^{2}}\left(tr{\left(\mu \right)}^{3}-27\right)+\ldots \right]+p\left(\frac{C}{C_{0}}-1\right),$$

where $k_{B}T$ is the thermal energy, *N* is the number of Kuhn segments in the polymer chain and I is the identity tensor. The above energy functional is obtained from the polynomial form of the series expansion of the inverse Langevin function for non-Gaussian chains [42,43]. The quantity *p* is a Lagrange multiplier that enforces the material’s incompressibility condition $C/C_{0}-1=0$ with $C_{0}$ being the density of chains at the stress-free configuration. Since the mass of the chain population does not change with deformation, this condition essentially enforces a volumetric constraint on the polymer network. Using the second law of thermodynamics and the assumption of an isothermal process, the dissipation energy, $D=k_{d}\Delta \Psi_{e}$, and the Cauchy stress tensor, σ, is given by

(6) $$\begin{array}{ccc} \sigma & = & ck_{B}T\left[\left(\mu -I\right)+\frac{6}{10N}\left(\lambda_{rms}^{2}\mu -I\right)+\frac{594}{1050N^{2}}\left(\lambda_{rms}^{4}\mu -I\right)\right]. \end{array}$$

The dissipation energy expresses the fact that chain dissociations in a stressed state result in overall loss of energy of the network, which cannot be recovered. The term D is equal to the rate of release of elastic energy due to chain dissociation. In other words, the polymer network undergoes relaxation by chain dissociation.

## 3. Flow Regimes under Steady Deformation Rates

### 3.1. Extensional Viscosity

Let us first consider a steady uniaxial elongational flow, where the strain in one direction increases at a constant rate in time. We now investigate the behavior of transient polymer networks subjected to such a macroscopic shear strain rate, $\dot{\gamma }$. This type of loading is typical of the rheological experiments used to measure “Trouton” viscosity [44] of polymer melts. Since the polymer network is incompressible, the sum of the diagonal components of the velocity gradient, tr(L)=0. Therefore, we can write the velocity gradient tensor as

(7) $$\begin{array}{c} L=\left(\begin{array}{ccc} \dot{\gamma } & 0 & 0\\ 0 & -\dot{\gamma }/2 & 0\\ 0 & 0 & -\dot{\gamma }/2 \end{array}\right). \end{array}$$

Physically, the above equation describes the deformation rate of a continuum point of the polymer network such that there is uniaxial tension in one direction and compression in the other two directions. The above deformation rate is applied on the polymer network that is initially at a stress-free state (σ=0). The stress reaches a steady value after some time, which is then used for calculating the viscosity of the network. Borrowing concepts from nonlinear viscoelasticity of non-Newtonian fluids, it is useful to introduce the non-dimensional Weissenberg number (*W*) which is a measure of the relative elastic to viscous forces. It is commonly written as the product of strain rate, $\dot{\gamma }$, and stress relaxation time $t_{r}=1/k_{d}$ [41]. For a steady extension rate, the stretch of the polymer chains in the network will eventually reach a state of dynamic equilibrium with the three physical processes (a), (b) and (c) described earlier. Therefore, the chain distribution function and tensor can be described by their steady state values, i.e., by substituting $\dot{\mu }=0$ in Equation (3b). We then get the chain distribution tensor (see Appendix A) as

(8) $$\begin{array}{c} \mu =\left(\begin{array}{ccc} 1/(1-2W) & 0 & 0\\ 0 & 1/(1+W) & 0\\ 0 & 0 & 1/(1+W) \end{array}\right),\;\;\;\;\;\;\mathrm{where}\;\;\;W=\frac{\dot{\gamma }}{k_{d}}. \end{array}$$

From the distribution tensor, we can extract the root mean square chain stretch ratio using Equation (2). To obtain a quantitative estimate of the chain orientation order, we consider the diagonal terms, $\mu_{1}$ and $\mu_{2}=\mu_{3}$, of the chain distribution tensor in Equation (8). Since they represent the standard deviation of the stretch ratio, λ, in each principal direction, the quantity $\Theta =|\mu_{1}-\mu_{2}|$ describes the magnitude of abundance in chain stretch in one direction compared to the other. When the polymer network is stress free, we have $\mu_{1}-\mu_{2}=0$, implying that the density of stretched chains is equally abundant in both directions, i.e., they are isotropic. Thus, the magnitude of stretch and the orientation of the attached chain population can be described by the quantities

(9) $$\begin{array}{c} \lambda_{rms}^{2}=\frac{1-W}{\left(1-2W)(1+W\right)}\;\;\;\;\;\;\mathrm{and}\;\;\;\;\;\;\Theta =\left|\frac{3W}{\left(1-2W)(1+W\right)}\right|. \end{array}$$

**Figure 2:**
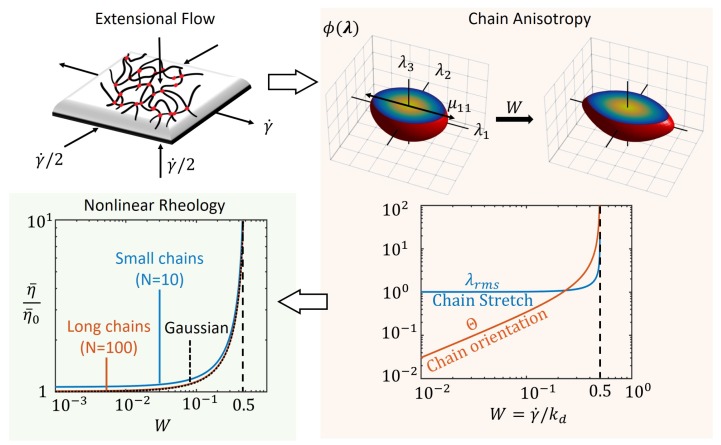
Steady extensional flow of a transient polymer network shown leading to chain anisotropy at steady state as seen from chain distribution function, ϕ(r) for different values of the Weissenberg number, *W*. The average chain stretch, $\lambda_{rmd}$, and orientation, Θ, are obtained from the chain distribution tensor, μ as a function of *W*. The nonlinear rheology is then captured by the plot of non-dimensional viscosity $\bar{\eta }/{\bar{\eta }}_{0}$, where $\bar{\eta_{0}}=3ck_{B}T/k_{d}$ is the viscosity of the network at zero extension rate.

We see that the above quantities diverge as W→0.5 as shown in the plots of Figure 2, indicating that the chains are highly stretched and oriented in the direction of tension. In other words, when the applied extensional rate, $\dot{\gamma }$ approaches the rate of bond dissociation, $k_{d}$, the chains do not have time to dissipate energy by dissociation and therefore end up storing elastic energy in their deformation. Also shown in Figure 2a are the contours of the distribution function ϕ(λ) obtained by numerically solving Equation (3a) with $\dot{\phi }=0$. The contours reflect the behavior of the entire chain population that transitions from an isotropic network (sphere) at no deformation to a highly oriented network (ellipsoid) at high extension rates. The statistical properties of this distribution, i.e., the standard deviation, is well captured by the chain distribution tensor as seen from Equation (8).

Using the steady state chain distribution tensor from Equation (8) and the Cauchy stress from Equation (6), the extensional (Trouton) viscosity $\bar{\eta }$ is obtained as

(10) $$\bar{\eta }=\frac{\sigma_{11}}{\dot{\gamma }}=\frac{3ck_{B}T}{k_{d}}\left(\frac{1}{\left(1-2W)(1+W\right)}\right)\left(1+\frac{6\lambda_{rms}^{2}}{10N}+\frac{594\lambda_{rms}^{4}}{1050N^{2}}\right)\;\;\;\mathrm{where}\;\;\;\;c=C\frac{k_{a}}{k_{a}+k_{d}}.$$

We see that the viscosity is also an increasing nonlinear function of the applied shear rate, $\dot{\gamma }$, and diverges when W→0.5 as plotted in Figure 2. This result is also consistent with that from standard polymer theory based on a coil-stretch transition [23] as $k_{d}$ here is the relaxation time of the network. Furthermore, we see that the effect of nonlinear chain elasticity does not play a significant role in the extensional viscosity as illustrated with small and long Langevin chains (different values for number of Kuhn segments *N*) and Gaussian chains. This is in part because of the geometric nature of this type of flow where parallel material planes separate exponentially with time as shown in the early work of Lodge for non-Newtonian behavior of “elastic” liquids. [45]. We have now shown this standard rheological result with the contribution of the value of the chain distribution tensor that contains information of anisotropy in the chain population for this type of flow.

### 3.2. Steady Shear Viscosity

We now investigate the behavior of transient polymer networks subjected to a macroscopic simple shear strain at a steady rate, $\dot{\gamma }$. This type of loading is more common in the rheological characterization of polymers and is used to measure the shear viscosity. Let us consider the shear loading in the 1–2 plane such that the velocity gradient tensor is given by

(11) $$\begin{array}{c} L=\left(\begin{array}{ccc} 0 & \dot{\gamma } & 0\\ 0 & 0 & 0\\ 0 & 0 & 0 \end{array}\right). \end{array}$$

Following the same approach from the previous subsection, we get the steady state chain distribution tensor (see Appendix A) as

(12) $$\begin{array}{c} \mu =\left(\begin{array}{ccc} 1+2W^{2} & W & 0\\ W & 1 & 0\\ 0 & 0 & 1 \end{array}\right). \end{array}$$

**Figure 3:**
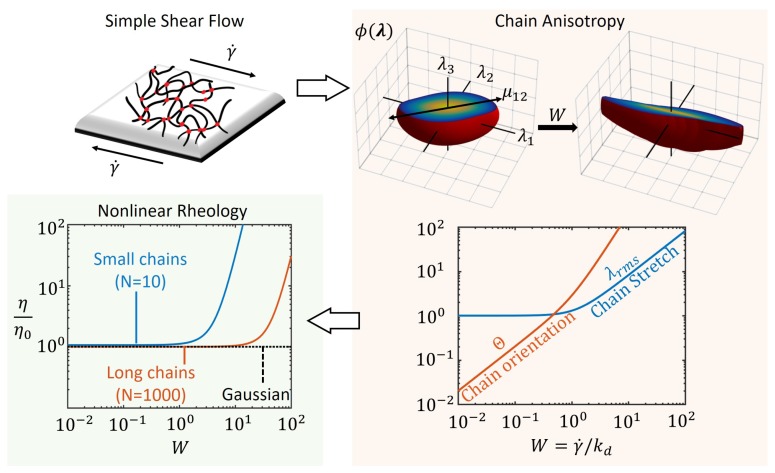
Steady shear flow of a transient polymer network shown leading to chain anisotropy at steady state as seen from chain distribution function, ϕ(r) for different values of the Weissenberg number, *W*. The average chain stretch, $\lambda_{rmd}$, and orientation, Θ, are obtained from the chain distribution tensor, μ as a function of *W*. The nonlinear rheology is then captured by the plot of non-dimensional viscosity $\eta /\eta_{0}$, where $\eta_{0}=ck_{B}T/k_{d}$ is the viscosity of the network at zero shear.

Interpreting the above tensor as the covariance matrix for statistical chain end-to-end vectors λ, we note that the chains align themselves in the direction of shear (1–2) as shown in Figure 3 with plots of the numerically computed distribution function ϕ(λ). The steady state value of the shear component $\mu_{12}$ is directly proportional to the Weissenberg number *W* implying that the chain stretch is maximum for large values of *W*. The flow of network may be described by the deformation of a continuum point of the polymer network with tension and compression given by the principle values in the corresponding principal directions. These principal directions of deformation rotate in time for a steady simple shear flow as is known from the classical concepts in continuum mechanics [45]. Accordingly, the principal directions of the chain distribution tensor also change during shear flow as can be shown from the eigendirections of Equation (12). However, the principal values ($\mu_{1},\mu_{2}$ and $\mu_{3}$) of μ can be determined to be constant in time for a given $\dot{\gamma }$, whose sum provides the mean square chain stretch ratio by Equation (2). Following the same definition for chain orientation order as the previous section, $\Theta =|\mu_{1}-\mu_{2}|$, the average chain properties during steady shear flow are

(13) $$\begin{array}{c} \lambda_{rms}^{2}=1+\frac{2}{3}W^{2}\;\;\;\;\;\;\mathrm{and}\;\;\;\;\;\;\Theta =2W\sqrt{1+W^{2}}. \end{array}$$

While the chain stretch increases linearly for large values of *W*, the orientation has a quadratic dependence on *W* as shown in the plot of Figure 3. This indicates that the chains become anisotropically oriented in the direction of shear, faster than the rate at which they are stretched. Due to dissipation by dissociation, the chain stretch and orientation are low for slow shear rates relative to the dissociation rate $k_{d}$. The chain distribution function ϕ becomes distorted more at large values of *W* and reflect the anisotropy in the chain population in terms of their direction of stretch (see Figure 3).

The macroscopic viscosity of the polymer network in steady shear is obtained from the shear stress $\sigma_{12}$ using Equations (6) and (12) as

(14) $$\eta =\frac{\sigma_{12}}{\dot{\gamma }}=\frac{ck_{B}T}{k_{d}}\left(1+\frac{6\lambda_{rms}^{2}}{10N}+\frac{594\lambda_{rms}^{4}}{1050N^{2}}\right)\;\;\;\mathrm{where}\;\;\;\;c=C\frac{k_{a}}{k_{a}+k_{d}}.$$

We see that the viscosity is also an increasing nonlinear function of the applied shear rate, $\dot{\gamma }$, for non-Gaussian chains while it remains a constant for Gaussian chains as plotted in Figure 3. Thus, the shear thickening behavior in transient polymer networks in this approach is attributed to the non-Gaussian nature of the chains, i.e., the chain tension increases drastically as the chain length approaches its maximum value (Nb). Furthermore, we see the effect of the maximum chain length on the onset of shear thickening as illustrated with small and long chains (different values for number of Kuhn segments *N*) with the latter requiring a larger shear rate for thickening. Based on Equation (14), the viscosity is a polynomial in terms of the shear rate $\dot{\gamma }$ whose coefficients are functions of the number of chain segments *N* and kinetic rates, $k_{a}$ and $k_{d}$. At the limit of small *W*, the fluid behavior is Newtonian as the viscosity is approximately a constant. Comparing Equations (10) and (14), we note that $\bar{\eta }\to 3\eta$, where factor 3 is the well-known Trouton ratio for Newtonian fluids [38,44].

The relationships obtained in Equations (13) and (14) show that the Weissenberg number, *W* determines the state of the chain population and emerging viscosity of the network at different shear rates. Though both mechanisms of non-Gaussian chain stretching and conversion of intra-chain to inter-chain cross-linking have been used to explain the shear thickening phenomenon [29,46,47], we propose that the above approach using the chain distribution tensor, is simpler and more informative in terms of the statistical measures such as chain stretch and orientation (anisotropy). It is our belief, therefore, that future studies should take into consideration the above approach to better understand and interpret nonlinear rheology and mechanics of transient polymers.

### 3.3. Network Fracture at Critical Strain Rates

In most transient polymer networks, it is observed that, with increasing shear rates, the shear thickening phenomenon eventually leads to a maximum viscosity after which catastrophic network fracture and subsequent reduction in viscosity (shear-thinning) occur [31,48]. This phenomenon has been attributed to a critical chain force, $f_{c}$, in the order of pN, when bonds start dissociating at an accelerated rate leading to increased dissipation of energy, faster relaxation and reduced viscosity [49]. In this section, we provide a relationship between the critical chain force, $f_{c}$ and the critical point of transition, ${\dot{\gamma }}_{c}$, when shear thickening switches to shear thinning. For this, let us first consider the chain stretch ratio, $\lambda_{c}$, corresponding to the critical force, $f_{c}$, given by the Langevin chain model as [50]

(15) $$\lambda_{c}=\sqrt{N}\left(\coth\left(\frac{f_{c}b}{k_{B}T}\right)-\frac{k_{B}T}{f_{c}b}\right).$$

**Figure 4:**
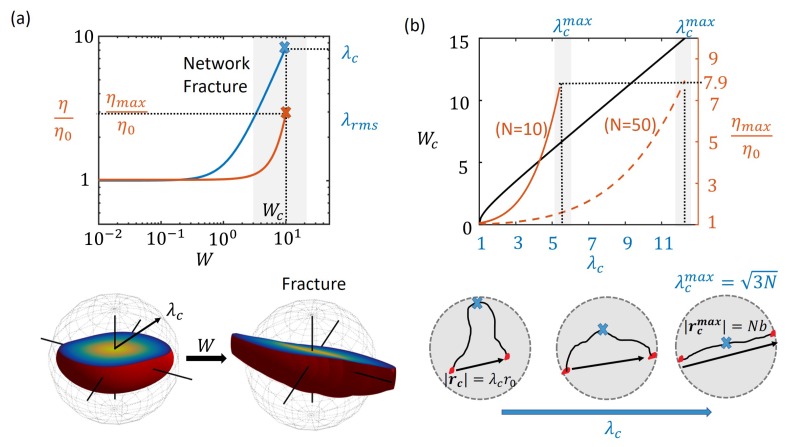
(**a**) the point of network fracture shown in a plot of viscosity η vs. Weissenberg number *W* when the average chain stretch equals the critical value, $\lambda_{c}$, for N=50. The distribution function is shown at steady state indicating most chains within the critical stretch at low shear rates (left) and leading to fracture at high shear rates (right); (**b**) the relationship between macroscopic entities such as maximum viscosity, $\eta_{max}/\eta_{0}$ and the critical Weissenberg number, $W_{c}$, and the critical chain stretch for fracture, $\lambda_{c}$ are plotted where the maximum value for the critical stretch ratio, $\lambda_{c}^{max}$ corresponds to that of a fully extended chain (r=Nb).

Network fracture occurs when the number of cross-links in the polymer network are suddenly reduced to below the gel point. Because high chain stretches lead to accelerated dissociation of cross-links, just before the point of network fracture, a majority of chains have a stretch above the critical value $\lambda_{c}$. In other words, the critical point for network fracture may be defined based on the root mean square stretch as $\lambda_{rms}=\lambda_{c}$. By considering the state of polymer chains in the steady state for different shear rates, a critical shear rate ${\dot{\gamma }}_{c}$ can be found where the average chain stretch ratio reaches the critical value (see Figure 4a). At this point, the viscosity of the network reaches its maximum value, $\eta_{max}$, which provides information on the extent of shear thickening in a given polymer network.

Based on the transient network theory applied to the steady shear rate loading from the previous section, we can obtain a relationship between the critical shear rate and critical chain force. This relationship can potentially help characterize the rheological behavior of different polymers on the molecular level in terms of chain properties. Substituting for $\lambda_{c}$ from Equation (15) in Equations (13) and (14) allows us to obtain the critical shear rate, $\dot{\gamma_{c}}$, and maximum viscosity, $\eta_{max}$, as

(16) $$\begin{array}{c} \dot{\gamma_{c}}=k_{d}\sqrt{\frac{3}{2}\left(\lambda_{c}^{2}-1\right)}\;\;\eta_{max}=\frac{ck_{B}T}{k_{d}}\left(1+\frac{6\lambda_{c}^{2}}{10N}+\frac{594\lambda_{c}^{4}}{1050N^{2}}\right). \end{array}$$

Firstly, based on the model assumptions, in simple shear flow, the polymer chains are stretched affinely at the applied shear rate and so the critical shear rate for fracture $\dot{\gamma_{c}}$ has a linear dependence on $\lambda_{c}$. Secondly, since shear thickening occurs in the present model due to non-Gaussian chain property whose tension, *f* increases rapidly with stretch λ, the maximum value of the viscosity $\eta_{max}$ also increases rapidly with large values of $\lambda_{c}$ for a given chain length *N*. Based on Equations (15) and (16), relative maximum viscosity of $\eta_{max}$ is only a function of the critical chain force $f_{c}$. Therefore, the value of $\eta_{max}$ for long chains is much lower for long chains (high *N*) than short chains (low *N*) for the same critical chain stretch, $\lambda_{c}$ (see Figure 4). Now, given that the maximum chain length is its contour length Nb, the maximum values of chain stretch cannot exceed $\lambda_{c}^{max}=\sqrt{3N}$. In reality, it is highly likely that a chain will dissociate with a very high probability when fully stretched as the elastic energy will overcome the energy barrier of the bonds [29]. Thus, we find from Equation (16) that the maximum value of viscosity during shear thickening cannot exceed roughly 7.9 times the zero shear viscosity $\eta_{0}=ck_{B}T/k_{d}$ (see Figure 4). Furthermore, when $\lambda_{c}^{max}$ is substituted in Equation (16), the maximum shear rate approximatey scales as $\dot{\gamma_{c}}\approx k_{d}N^{1/2}$ consistent with the finding by Marrucci et al [34] with the “free path” model.

Therefore, we conclude that, while the main role of the critical chain stretch $\lambda_{c}$ is in determining the maximum possible shear rate ${\dot{\gamma }}_{c}$ before network fracture, the extent of shear thickening is determined by the critical chain force $f_{c}$. The above relationship may be useful in estimating the critical chain stretch from measured macroscopic properties of the network such as maximum viscosity and critical shear rate or vice versa.

### 3.4. Viscosity at High Cross-Linker Densities

**Figure 5:**
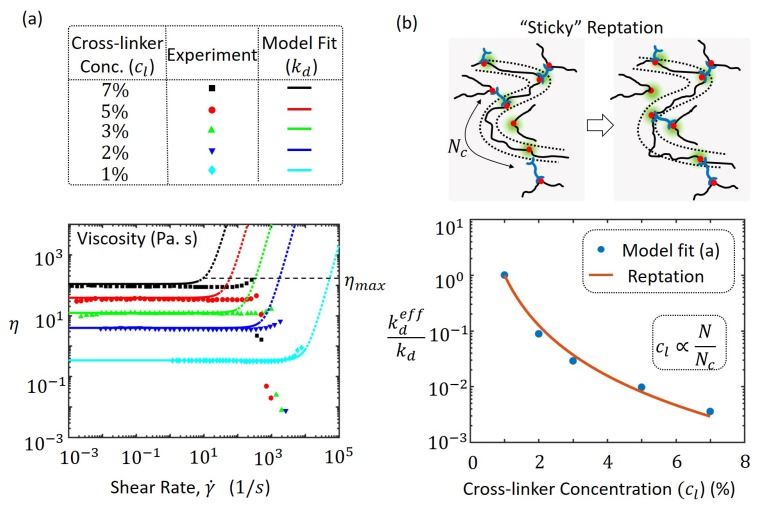
(**a**) experiment and model prediction of poly(4-vinylpyridine) (PVP) cross-linked with bis-Pd(II) complex for different cocncentration of cross-linkers [29]. The dotted lines for the model prediction indicate viscosity beyond the failure point with maximum viscosity $\eta_{max}$; (**b**) illustration of the “sticky” reptation mechanism at large concentrations with constrained chain movement in a tube with intermittent cross-linkers. Shown below is the plot of effective dissociation rates, $k_{d}^{eff}$ obtained from fitting the experimentally measured viscosities of (**a**) and prediction of the reptation model

In addition to its dependence on applied shear rate, the rheology of polymer networks with reversible bonds can be significantly affected by the density of cross-linkers present. For example, with metallo-supramolecular networks of poly(4-vinylpyridine) (PVP) in dimethyl sulfoxide (DMSO), the concentration of reversible bis-Pd(II) cross-linkers altered the measured viscosity [29] as shown in Figure 5a. The cross-linkers contain a metallic(Pd) complex that can form a reversible ligand interaction with the PVP polymer chain that contains pyridine nitrogens. Therefore, the concentration of cross-linkers reported in terms of molar ratios between palladium and pyridine nitrogen is directly correlated to the cross-linking density of this transient polymer network. The viscosity measurements indicate an increasing trend with higher cross-link densities as shown in Figure 5a, which have been fitted with our model using Equation (10) with chain concentration, *C*, number of Kuhn segments in the chain *N* and different values of $k_{d}$ for the varying cross-linker concentration. We have here assumed that the rate of bond association, $k_{a}$ is the same as the rate of bond dissociation, i.e., bond dissociations and associations occur at the same time scale. An inverse correlation of the concentration of cross-linkers, $c_{l}$, with the number of Kuhn segments $N_{c}$ in the effective chain, and the rate of dissociation, $k_{d}$, affect the base viscosity $\eta_{0}$ and the onset of shear thickening according to the model. This is because the Weissenberg number dictates shear thickening as seen in Equation (14), which for a lower $k_{d}$ requires a lower shear rate, $\dot{\gamma }$ for the same value of *W*. The onset of shear thickening for different concentrations of cross-linkers, qualitatively follows the same trend as experiments that is consistent with the argument based on the Weissenberg number. The average rate $k_{d}$ is also the stress relaxation rate in transient polymer networks that have relatively low variation in their bond lifetimes across the material. The extent of shear thickening described by the maximum viscosity, $\eta_{max}$, is determined from experiments and using Equation (16), the non-dimensional critical chain force is determined from Equations (15) and (16) to have an average value of $f_{c}b/k_{B}T=4.1$. The disagreement in the onset of shear thickening between the model and experiment could be possibly due to a spectrum of relaxation rates such that of Rouse modes from chain entropy or force-sensitive chain dissociation that have not been addressed with the current model. The mismatch is especially apparent for high cross-linker concentrations where the effective number of chain segments $N_{c}$ is lower which thereby increases the Rouse relaxation rate due to its inverse relationship with $N^{2}$ [50]. Similarly, since the chain tension increases rapidly for small chains a force-dependent chain dissoication rate would be highly significant for high cross-linker concentrations. Both these phenomena tend to increase the net relaxation rate and faster energy dissipation of the polymer network thereby requiring a larger shear rate $\dot{\gamma }$ to reach the same Weissenberg number *W*. These phenomena and the mechanism of conversion of intra-chain to inter-chain cross-linkers discussed and identified as the dominant cause of shear thickening in these supramolecular networks [29] may be better addressed in detail in future studies. The approach introduced in this paper through the chain distribution tensor will be highly useful in addressing such concerns.

To explain the inverse correlation between the dissociation or relaxation rate, $k_{d}$, and the cross-linker concentration, $c_{l}$, we propose a mechanism that is closely related to the so-called “sticky” reptation mechanism introduced by Leibler et al. [18] for entangled polymer networks with reversible cross-links. When long polymer chains are introduced with cross-linkers, a polymer network is formed at a critical concentration and is called the gel point [50]. In this structure, polymer chains are connected or cross-linked to other chains mostly at their two ends. When the concentration of cross-linkers is increased, more connections are likely at interior points of the chains causing the chain to be more restricted in terms of its dynamics and motion (see Figure 5b). Let us consider a monodisperse chain of *N* monomers that has an average end-to-end distance of $b\sqrt{N}$, where *b* is the Kuhn length of a monomer segment. Due to high concentration of cross-linkers, let the average number of monomers along the chain between two cross-linkers be denoted by $N_{c}$ that is less than *N*. The number of cross-linkers per chain is then given by $N/N_{c}$ and is directly proportional to the cross-linker concentration, $c_{l}$. As the chain is restricted to move in a tube due to constraints from the presence of additional cross-linkers, the average contour length, <L>, of the chain is the sum of the average end-to-end distance between cross-linkers each of which is $b\sqrt{N_{c}}$. This is given by

(17) $$\begin{array}{c} <L>=\frac{N}{N_{c}}\;b\sqrt{N_{c}}=\frac{N}{\sqrt{N_{c}}}b. \end{array}$$

To obtain the relaxation time in networks with chains of the above type, let us consider the dynamics of chain motion in a constrained tube created by intermittent cross-linking. Similar to the reptation model for entangled chains, the chain motion consists of diffusion through small loops along the contour length. The reptation time, $\tau_{r}$, of the chain depends directly on the square of the chain length, $<L{>}^{2}$, in Equation (17), and inversely on the Rouse diffusion coefficient, $D_{c}=k_{B}T/\zeta$, where ζ is the dynamic friction coefficient. The quantity ζ can be obtained from a force–velocity relationship for the chain based on the concepts of the transient network theory in 1D (see Appendix B) as

(18) $$\begin{array}{c} \zeta =\frac{k_{B}T}{N_{c}b^{2}}\left(\frac{N}{N_{c}}\right)\frac{1}{k_{d}}. \end{array}$$

The relaxation rate of the polymer network at high cross-link density is given by the time required by the chain to reptate, $\tau_{r}=<L{>}^{2}/D_{c}$, through the tube since it is slower than the bond dissociation rate, $k_{d}$. Therefore, the effective relaxation rate is given by $k_{d}^{eff}=1/\tau_{r}$. Using Equations (17) and (18) and after simplification, we obtain

(19) $$\begin{array}{c} k_{d}^{eff}={\left(\frac{N_{c}}{N}\right)}^{3}k_{d} \end{array}$$

that predicts an effective dissociation rate that is slower when the number of chain segments between cross-links, $N_{c}$ is less than that for the entire chain, *N*. In other words, when the cross-linker concentration, $c_{l}$, is increased, there are more intermittent cross-linking causing an increase in apparent “chain friction” and a slower relaxation rate. A low effective dissociation rate, $k_{d}^{eff}$, for high cross-linker concentration when applied to Equation (10), predicts a higher viscosity that explains the experimental measurements shown in Figure 5a. The fit from the model in (a) is compared with prediction from the reptation model from Equation (19) with good agreement in Figure 5b.

## 4. Conclusions

In summary, we have provided a quantitative interpretation of nonlinear rheology in transient polymer networks in terms of chain stretch distributions rooted in a statistical description of polymer chains, bond association and dissociation. We particularly addressed three main phenomena commonly observed, namely (a) the nonlinear increase in viscosity for two different regimes namely steady extensional and shear flows (shear thickening) with increase in deformation rate, (b) network fracture at extremely large shear rates preceding reduced viscosity or shear-thinning, and (c) increased viscosity due to high cross-linker density due to slow chain diffusion. The phenomenon of shear thickening is found to be closely related to the Weissenberg number consistent with previous interpretations regarding polymer networks [23]. Furthermore, the effect of non-Gaussian chain behavior is shown to have an significant role in the onset of shear thickening assuming that it is the driving mechanism. We have provided a simple relationship between the critical shear rate for network fracture and the critical chain force for breakage that is made possible through the transient network theory. An important contribution of this work is the relationship of chain properties such as stretch and orientation and macroscopic properties such as viscosity through the chain distribution tensor. This tensor provides a statistical measure of the chain population and helps provide a clearer picture of the mechanisms involved. Finally, the increase in viscosity at high cross-link concentration is explained by a slow down of chain diffusion occurring from constrained chain dynamics akin to a “sticky” reptation model with good agreement with experimental measurements. The effect of reduced viscosity or shear thinning beyond the critical shear rate, though not addressed here, could be investigated further in future work with the current framework using a dissociation rate that is accelerated by force.

## Abbreviations

The following abbreviations are used in this manuscript:

TNT	Transient Network Theory
MDPI	Multidisciplinary Digital Publishing Institute
DOAJ	Directory of Open Access Journals

## Appendix A. Derivation of Solution for Shear Thickening Equations

Consider the evolution equation of the distribution tensor, Equation (3b). For a steady elongational flow rate given by the velocity gradient in Equation (7), the network reaches a steady state at which the evolution equations reduce to

(A1) $$\begin{array}{ccc} k_{a}\left(C-c\right)-k_{d}c\mu_{1}+2c\dot{\gamma }\mu_{1} & = & 0,\\ k_{a}\left(C-c\right)-k_{d}c\mu_{2}-c\dot{\gamma }\mu_{2} & = & 0,\\ k_{a}\left(C-c\right)-k_{d}c\mu_{3}-c\dot{\gamma }\mu_{3} & = & 0, \end{array}$$

where $c=Ck_{a}/\left(k_{a}+k_{d}\right)$ and $\mu_{1}$, $\mu_{2}$ and $\mu_{3}$ are the diagonal terms of the chain distribution tensor while the non-diagonal terms are zero. Solving the above equations, these components are obtained as

(A2) $$\begin{array}{c} \mu_{1}=\frac{1}{1-2W}\;\;\;,\;\mu_{2}=\frac{1}{1+W}\;\;\;\mathrm{and}\;\;\;\mu_{3}=\frac{1}{1+W}, \end{array}$$

where $W=\dot{\gamma }/k_{d}$.

For a steady shear flow rate given by the velocity gradient in Equation (11), the network reaches a steady state at which the evolution equations reduce to

(A3) $$\begin{array}{c} W\left(\begin{array}{ccc} 2\mu_{12} & \mu_{22} & \mu_{23}\\ \mu_{22} & 0 & 0\\ \mu_{23} & 0 & 0 \end{array}\right)+\left(\begin{array}{ccc} 1-\mu_{11} & -\mu_{12} & -\mu_{13}\\ -\mu_{12} & 1-\mu_{22} & -\mu_{23}\\ -\mu_{13} & -\mu_{23} & 1-\mu_{33} \end{array}\right)=0. \end{array}$$

Solving the above the equation for each component, we obtain

(A4) $$\begin{array}{c} \mu_{11}=1+2W^{2},\\ \mu_{22}=\mu_{33}=1,\\ \mu_{12}=W,\\ \mu_{13}=\mu_{23}=0. \end{array}$$

Using Equation (6), the Cauchy stress σ may then be obtained from the chain distribution tensors derived above for both types of flows.

## Appendix B. Derivation of Friction for “Sticky” Reptation Model

The friction acting on a polymer chain that is reptating through a tube with intermittent cross-linking is determined by reducing the chain population to one dimension (along the tube). The polymer chain may thus be treated as an assembly of smaller chains which have their own corresponding stretches. The total number of such segments is given approximately by $n_{x}=N/N_{c}$. We note that the quantity c(t), the density of attached tethers in 3D, becomes $n_{a}$, the number of detached tethers in 1D. We also observe that the velocity gradient tensor L reduces to $\dot{\delta }/\delta$ in 1D, with δ being the chain end-to-end distance and $\dot{\delta }=v$, the velocity of the chain. We can then arrive at the evolution equation using Equation (3a) as

(A5) $$\frac{\partial \phi (\delta ,t)}{\partial t}=k_{a}N\left[1-n\left(t\right)\right]p_{0}\left(\delta \right)-k_{d}\phi \left(\delta ,t\right)-v\frac{\partial \phi (\delta ,t)}{\partial \delta }.$$

If $p_{0}\left(\delta \right)$ is assumed to be a Dirac delta function centered at δ=0, i.e., all tethers re-attach in the stress-free configuration with zero displacement, we find that an equivalent expression to

(A6) $$\frac{\partial \phi (r,t)}{\partial t}=k_{a}\left[C-c\left(t\right)\right]p_{0}\left(r\right)$$

is, in 1D,

(A7) $$v\phi \left(0,t\right)=k_{a}\left[n_{x}-n_{a}\left(t\right)\right].$$

Using the above result, an equivalent formulation for Equation (A5) is the reaction– advection equation

(A8) $$\frac{\partial \phi (\delta ,t)}{\partial t}=-k_{d}\phi \left(\delta ,t\right)-v\frac{\partial \phi (\delta ,t)}{\partial \delta }$$

with boundary condition at δ=0:

(A9) $$\phi \left(0,t\right)=\frac{k_{a}}{v}\left[n_{x}-n_{a}\left(t\right)\right].$$

The total force on the long chain is given by the summation of forces on each individual segment and can be written as

(A10) $$F=K\int_{0}^{\infty }\phi \left(\delta ,t\right)\delta d\delta ,$$

where $K=k_{B}T/N_{c}b^{2}$ is the stiffness of the chain segment with $N_{c}$ monomers in 1D. Now, we transform the integral equation for total force to a differential equation. We have:

(A11) $$\begin{array}{cc} \dot{F} & =\frac{d}{dt}K\int_{0}^{\infty }\phi \left(\delta ,t\right)\delta d\delta \\ & =K\int_{0}^{\infty }\frac{\partial \phi }{\partial t}\left(\delta ,t\right)\delta d\delta \\ & =K\int_{0}^{\infty }\left[-k_{d}\phi -v\frac{\partial \phi }{\partial \delta }\right]\delta d\delta \\ & =-k_{d}K\int_{0}^{\infty }\phi \delta d\delta -Kv\left(\phi \delta {|}_{0}^{\infty }-\int_{0}^{\infty }\phi d\delta \right)\\ & =-Kv\left(0-n_{a}\right)-k_{d}K\left(\int_{0}^{\infty }\phi \delta d\delta \right)\\ & =Kvn_{a}-k_{d}F. \end{array}$$

To obtain the dynamic friction coefficient, we assume a constant force *F* such that $\dot{F}=0$ to give

(A12) $$F=\frac{Kn_{a}}{k_{d}}v=\zeta v.$$

Substituting for *K* in the above expression and $n_{a}=N/N_{c}$ for the number of intermittent cross-links, we get the friction coefficient as

(A13) $$\zeta =\frac{k_{B}T}{N_{c}b^{2}}\left(\frac{N}{N_{c}}\right)\frac{1}{k_{d}}.$$
